# An Alternative Approach to Protein Folding

**DOI:** 10.1155/2013/583045

**Published:** 2013-09-02

**Authors:** Yeona Kang, Charles M. Fortmann

**Affiliations:** Department of Materials Science and Engineering, Stony Brook University, Stony Brook, NY 11794-2275, USA

## Abstract

A diffusion theory-based, all-physical *ab initio* protein folding simulation is described and applied. The model is based upon the drift and diffusion of protein substructures relative to one another in the multiple energy fields present. Without templates or statistical inputs, the simulations were run at physiologic and ambient temperatures (including pH). Around 100 protein secondary structures were surveyed, and twenty tertiary structures were determined. Greater than 70% of the secondary core structures with over 80% alpha helices were correctly identified on protein ranging from 30 to 200 amino-acid sequence. The drift-diffusion model predicted tertiary structures with RMSD values in the 3–5 Angstroms range for proteins ranging 30 to 150 amino acids. These predictions are among the best for an all *ab initio* protein simulation. Simulations could be run entirely on a desktop computer in minutes; however, more accurate tertiary structures were obtained using molecular dynamic energy relaxation. The drift-diffusion model generated realistic energy versus time traces. Rapid secondary structures followed by a slow compacting towards lower energy tertiary structures occurred after an initial incubation period in agreement with observations.

## 1. Introduction

This work introduces an *ab initio* physical drift and diffusion-based protein structure prediction simulation that runs on a desktop PC. The protein folding dynamic is one of the most important problems in biology (e.g., see [1, 2]). Given the numerous manuscripts, journals, and volumes that have been dedicated to the techniques, the progress, and the importance of this work, it is not possible to give fair review here. That being said, the authors recognize the fantastic progress made in statistical and homolog based approaches and the advances these have contributed to all aspects of protein science. However, there are many cases where appropriate homologs are not available and/or where protein structure must be predicted in environments that differ markedly from those used to obtain homolog experimental structures. Also, many protein folding simulations such as molecular dynamics (MD) require large amounts of CPU time for protein structure folding and/or prediction and require templates (or homologs) for initiation [3]. For cases without appropriate homologs, an *ab initio* model with reasonable accuracy is valuable.

Several methods to assess the performance of protein structure prediction have evolved. One is the testing of a sufficient number of cases to demonstrate the performance of a given approach. Another is the (CASP) Critical Assessment of Techniques for Protein Structure Prediction competition [4]. CASP scores the various models by comparing experimentally discovered structures to those obtained by the competition organizers. This work employed CASP9 as a template-free or *ab initio* modeling [4] and was ranked among the middle scoring groups.

Here, a protein folding and structure prediction model based on the first principle forces (energy gradients) and physical kinetics including the drift and diffusion of residues and/or protein substructures relative to one another, is described.

## 2. Theoretical Backgrounds

### 2.1. Multiple Energy Considerations

 Physical kinetics and the near equilibrium descriptions are fundamental to chemical engineering and materials science (see, e.g., Barratt et al. [5] and Moore and Pearson [6]). Here, these principles are applied to the protein folding dynamic and the protein structure prediction. The underlying assumption being that the change from one ambient to another is carried out slowly enough to justify near equilibrium mechanics. Challenges include relative subprotein structures motion in two or more energy fields.

In steady state and near equilibrium, the diffusion-driven dispersion and force-driven drift must balance to preclude unnatural energy accumulations. In protein structure, two or more energy fields almost always simultaneously act. Resolving particle motion in two or more energy fields was previously described [7]. The work showed that relative motion induced in global protein entropy change could be incorporated into electric field mobility [7]:

(1) $$\begin{array}{c} \mu =\frac{D\nabla \ln\left(n_{p}\right)}{\nabla \left\{VP-ST+\sum_{j}\phi_{L_{j}}n_{j}\right\}+kT\nabla \ln\left(n_{p}\right)}, \end{array}$$

where *D* is the diffusivity, *k* is the Boltzmann constant, and *T* is temperature in Kelvin. It was found that only the largest energy changes (e.g., the product of global entropy and temperature) made significant contribution to this mobility where  *n*_*p*_  is an identifiable protein substructural species or atom concentration and the summation is over all species  *n*_*j*_  having a related (nonelectrostatic) energy (e.g., global entropy-temperature product or stain energy),  *ϕ*_*L*_*j*__, subject to change upon folding.

As ambient conditions change, the protein energy (or free energy) becomes greater than that at equilibrium. Relative energies and forces (gradients of the relative energy) act on each member of the protein. These forces in turn result in a drift speed defined by

(2) $$\begin{array}{c} \text{drift speed}=\mu F, \end{array}$$

where  *F*  is the force on a particular atom or site and  *μ*  is the drift mobility (1).

### 2.2. Force Considerations

 While global entropy change was incorporated into mobility, it is necessary to consider the several other energy gradients (force) inducing motion. Forces were summed and subprotein structures were allowed to move relative to one another. In this work, the net charge and hydrophobicity of each side chain of residues were assigned to the nearest backbone atom (see Figure 4).

Four explicit forces were considered: the electrostatic (Coulomb's) force (applied to charges and dipoles), electrostatic displacement force (as defined below), generalized diffusive force (a thermal force as defined below), and global entropy change. Summed forces about each alpha carbon (in pivot bond) were cataloged, and the greatest was chosen to produce torque and motion about the identified alpha carbon atom. After the drift-diffusion determination of tertiary structure, the molecular dynamics (MD) model was used for final structure determination via energy relaxation.

The electrostatic force between two charged bodies (*q*_1_  and  *q*_2_) is easily determined from Coulomb's law using an appropriate dielectric constant (3). This research found that the dielectric constant of water, *ϵ*_*w*_, yielded good results, where  *ϵ*_*w*_  is ~78*ϵ*_0_ (where  *ϵ*_0_  is the permittivity of vacuum). Therefore, the Coulomb force is

(3) $$\begin{array}{c} F_{\text{elect}}=\frac{q_{1}q_{2}}{4\pi \epsilon_{w}r^{2}}. \end{array}$$

Kang et al. previously described the electrostatic displacement force in the context as used here [7] and as applied in neural ionic transport where the electrostatic displacement force arises from the attraction between mobile (e.g., liquid) polar media and an electric field [8]. The polar media are drawn towards an increasing electric field thereby producing a force that acts to sweep mobile uncharged, nonpolar media (e.g., fixed ion electric field, liquid water, and a noncharged residue) producing attraction. The attraction of polar media produces a corresponding displacement force that always moves nonpolar protein regions towards a lower electric field region (i.e., the nonpolar regions are pushed away from strong electric field by the inrush of water or other polar media toward strong field).

Simplified models for interaction of water, nonpolar media, and electric field with respect to protein structure have been developed. For example, the generalized born (GB) model [9] has been used to track the electric field energy and/or solvation energy with respect to protein charge and partial charge. Since this work follows a folding protein, the various protein charge and partial charge move with respect to one another. Therefore, of the cases examined by Bashford and Case [9], the case in which two proteins moved with respect to one another most closely relates. The kinetic model (KM) approach enabled a simple physical approach to these considerations (see Kang et al. [7]).

Water dielectric constant is reasonable for distant charged species since the predominant media separating these is water. However, in the case of secondary structure and collapsed protein structure, the small distances between charged species are likely to have significant protein content having a low dielectric constant. Nonetheless, the simulation produces secondary structure with a high degree of accuracy. In the secondary structure generating subroutine described in the next section, parameters are fine-tuned to produce the best agreement with experiment, and even higher accuracy is obtained. Typically, the highest accuracy results for the tertiary structure determination were obtained using the fine-tuned secondary structure subroutine to identify secondary structure location, followed by a KM simulation collapse of the denatured protein with secondary structures and finally a full molecular dynamic relaxation (based on AMBER10) of the 3D structure in which local dielectric property is generated and used. As noted elsewhere, the use of AMBER10 (and its more accurate dielectric consideration) increased accuracy by about  3%.

There is an energy associated with the electric field imbedded in a dielectric media. Forces can be generated when charge or nonpolar structure motion induces a reduction in this energy. Therefore, it is necessary to define a displacement energy and a displacement force described in terms of the electric field energy,  *W*, for water-filled regions (with permittivity  *ϵ*_*w*_) relative to the region filled with a nonpolar structure (assumed to have the permittivity of vacuum,  *ϵ*_0_).

**Figure 1:**
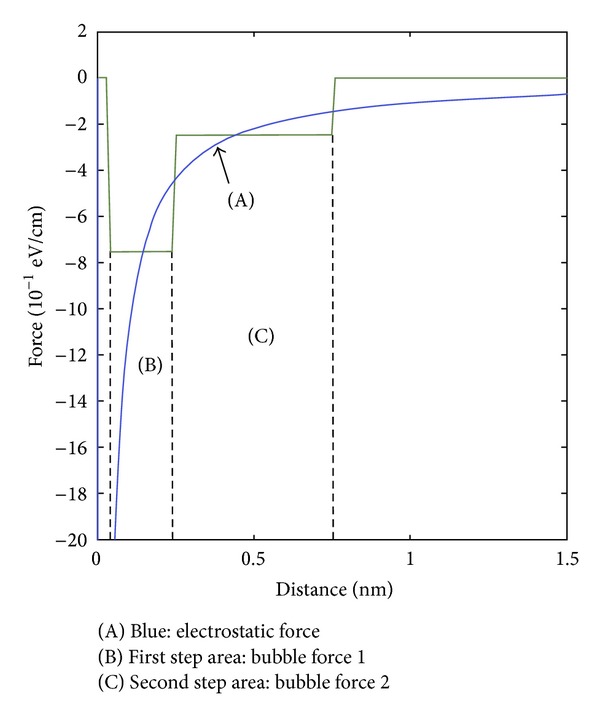
Displacement force: the electrostatic force (A), the force map representing direct contact of two nonpolar residues (B), and extension of displacement force (C) corresponding to conveyance of the displacement force via equilibrium nonpolar interaction.

Following Jackson [10], this energy consideration can be quantified:

(4) W=−12∫V1(ϵw−ϵ0)E→•E→0dV,

(5) ≈−12(ϵw−ϵ0)E→•E→0ΔV,

(6) ≈−β|q|2r4,

(7) Force=−∂W∂r=−4β|q|2r5,

where the integration is over the volume of the nonpolar protein region. The nonpolar volume and the other constants are collected into single term  *β*  (as seen in (6) and (7)) without loss of accuracy. The magnitude of the displacement forces acting upon a neutral alpha helix-sized object under the influence of an electronic point charge at ~0.1 nm is comparable to the Coulomb force between two opposite point charges at a similar distance.  Figure 1 shows the displacement force values based on the distance between two nonpolar regions relative to electrostatic force.

The third force considered here is thermal force. Thermal force is related to the average motion or speed of one part of the protein relative to another arising from diffusion. There are several possible formulations of the thermal force. The thermal diffusion speed is$\;\nu =\sqrt{D/\tau }\to \sqrt{\mu kT/\tau }\;$(since  *μ* = *D*/*kT*). The force needed to produce the same speed through drift is$\;\sqrt{kT/\mu \tau }\;$where  drift  speed = *μ*∇*ϕ*_eff_  and  ∇*ϕ*_eff_  is an effective force.

The concept of thermal force can be understood in the context of generalized force (e.g., see Glicksman [11]), whereby a concentration gradient and the corresponding diffusive currents offset an applied force. This principal is widely applied, for example, in the separation of isotopes, where the inverse, the force needed to halt a diffusion-driven concentration change, is considered.

The forth force, global entropy change, was considered. Here the global entropy is taken to be the aggregate by all protein entropy terms that change under folding. A change in global entropy is estimated by considering a proposed change in protein structure. The global entropy values used here (generally decreasing on compacting) were determined by fitting to observation (see Kang et al. [7]). The estimated entropy change was in turn used to determine a new mobility. A proposed protein change was computed by allowing a force to act, producing a speed consistent with the new mobility. The resultant speed was allowed to act for one time step.

Total force in protein structure can be defined by the summation of three forces (8). The forth force, global entropy-temperature product is incorporated into the mobility. The best structure predictions were obtained when the global entropy diminished on folding. For example, at room temperature an entropy-temperature gradient (2nd term in the denominator of (1)) of *≈*10^4^ eV/(Kcm) was typical.

The remaining three forces can be expressed as

(8) F=(q1q24πϵr2)+(β|q|2r5)+kTμτ,

(9) →(q1q24πϵr2)+(β|q|2r5)+γkT,

where defining the quantity$\;\sqrt{1/\mu \tau }\to \gamma \;$helps clean up the equations.

The relation among charge, water, and hydrophobic residue association is quantified in (9). The first term of (9) (Coulomb) quantifies the force between charges and/or charge and polar structure.

It is well known that hydrophobic residues aggregation and water surface reduction relate to alpha helix formation. These hydrophobic interactions are characterized in terms of the aforementioned displacement force. However, in careful examinations of natural protein structures, there exist many protein regions containing large numbers of hydrophobic (nonpolar) residues, which do not bond (aggregation) and do not form alpha helices. This is understood in terms of the displacement force whereby charge attracts polar water, and in turn, the polar water pushes nonpolar hydrophobic residues apart.

**Figure 2:**
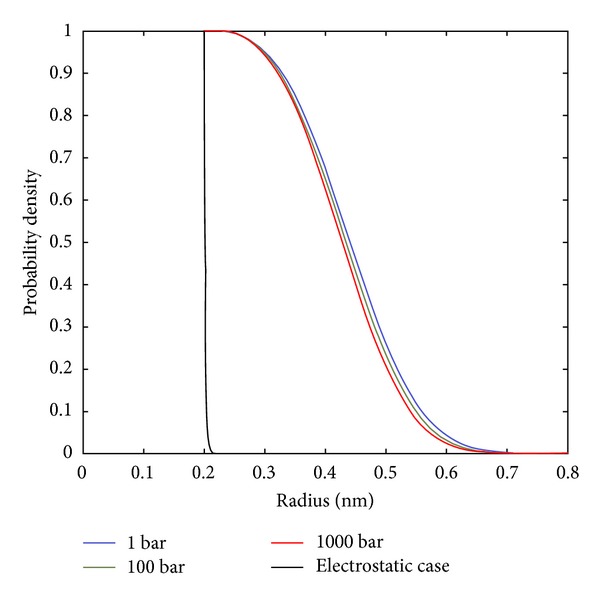
The probability of a nonpolar region in water as a function of pressure and electric field (as indicated) is shown. Increasing electric field significantly reduces the nonpolar region probability.

The repulsive force (second term of (9)) is pushing uncharged nonpolar structures away from the charged regions (by incoming polar water) causing the hydrophobic residues to remain separated. Therefore, when sufficient charge exists in the intervening protein segments (e.g., charged residues), the repulsive component (second term of (9)) generates a repulsive force sufficient to block the more outlying hydrophobic residues from approaching one another.  Figure 2 illustrates this electric field with induced blocking. Additionally, it was found that extremely large residue sizes could also inhibit alpha helix formation.

The thermal force tends to randomize the net force or torque acting on any given backbone atom pair. However, early in the folding, the strong force between nearly spaced hydrophobic and electrostatic pairs overwhelms the thermal force. Consequently, the simulation tends to hang up (repeatedly returns to move on) a particular strong force generating pair. After a particular atom pair generates the largest torque for five sequential time steps, the simulation freezes the motion about this particular backbone atom pair by removing its force (or torque) from the next time step query. Thereby, the simulation preserves the structure generated in five time steps relative to the torques generated by the nearly spaced hydrophobic and/or electrostatic pairs.

It was found that almost all of the frozen backbone atomic pairs were part of secondary structures and that these formed early in the folding process. In this regard, these structures are related to autonomous folding units [12]. In turn, the relationship between strong forces on nearly spaced hydrophobic and electrostatic pairs can be directly correlated to secondary structure as considered in the next section.

Equation (9) is useful on all size scales. On the smallest size scale, (9) can be the potential to generate secondary structure. On larger size scales, the expression guides the generation of tertiary structures by describing the force between charged regions and hydrophobic secondary structures. From the insights provided by this description, a set of rules governing secondary structure formation were developed.

One of the attributes of the presented physical model is that the workings that produce structure are open to inspection and understanding. Accordingly, examination of secondary structure, in some cases time step by time step, revealed the nearly balanced interplay of the hydrophobic versus electrostatic elements of (9). Consequently, two paths for improved secondary structure were presented. First, the conceptually simpler is the fine-tuning of the force equations. The second is that the recognition of nearly balanced hydrophobic and electrostatic forces imply secondary structure formation. Therefore, an algorithm was developed to search for large, nearly balanced force. In this writing, the second approach (described in the following section) is the more accurate. Using the second approach requires the use of a graphic program (such as Pymol) to insert the appropriate structure into the 3D structure.

## 3. A Streamlined Secondary Structure Prediction Model

 Historically, secondary structure prediction has advanced over the past five decades. Secondary structure prediction introduced in the 1960s and early 1970s [13–15] focused on identifying alpha helices. Beta sheet identification also relying on statistical analysis [14, 15] began in the 1970s. Evolutionary conservation methods exploited the simultaneous assessment of many homologous sequences to determine probabilistic relation between protein sequence and secondary structure [16, 17]. Larger experimental structure databases and modern machine learning methods have achieved ~80% overall secondary structure prediction accuracy in globular proteins [18].

In the presented model, the relative location of residues and the hydrophobic/polar and charge characteristics of each residue are the only input elements. The hydrophobic character as a function of ambient can be found in the literature (see, e.g., [19, page 14, Table 1.2]). Also, amino acid charge and partial charge can be determined using popular software suites including the (MOE) Molecular Operating Environment software package which employs the standard AMBER 10 parameter set to calculate the force field [20]. In some cases (not reported here), extreme ambients such as very low pH require appeal to more sophisticated commercial software suites.

The algorithm for secondary structure prediction systematically steps through an arbitrary amino acid sequence. When hydrophilic amino acids are encountered, a mechanical set of queries determines if secondary structure is present. These queries relate to the hydrophilic character of the following neighboring residues. The simulation used by this work employs secondary structure search beginning at the occurrence of a hydrophilic residue after hydrophobic residue and ending at the next hydrophobic residue. The charge, size, and polarity of the intervening amino acid residues determine both a secondary structure region and its type. A delicate balance of charge, polarity, and hydrophobic character determines the secondary structures of protein.

Upon encountering a hydrophilic amino acid (*n*_*i*_), a scan bracket is opened. The following in the sequence (*n*_*i*+1_,…, *n*_*i*+6_) are queried unless the finding of a hydrophilic residue ends the search (i.e., the end point (*n*_*i*+*j*_) occurs at the next hydrophilic residue). If no hydrophilic residues are encountered within the  *i* + 6  nearest neighboring residues, the algorithms denote the region as unstructured and then move on to the next hydrophilic residue in the sequence where a new search begins. Importantly, in all cases where a second hydrophilic residue (thus ending a search) is found within the next six nearest neighbors a secondary structure alpha helix will form in accordance with the rules summarized in Table 1.

Determination of the secondary structure type within a given sequence meeting the six nearest neighbors rule follows a simple hierarchy. First, if the stringent conditions for hydrophobic collapse are unopposed by (sufficiently large) dielectric displacement forces, an alpha helix will form. Second, whenever alpha helix formation is blocked (e.g., by the dielectric displacement force induced by intervening charged residue), a beta sheet region will form. Therefore, alpha helix regions can be transformed into a beta sheet by mutations resulting in one or more charged residue(s) in the critical areas between hydrophobic residues. The formation of fibrils in Alzheimer's patients is an example of a case where a small mutation causes an alpha helix collapse [21].

The summation of charges (Table 1, column  2) determines the overall charge, and therefore, the magnitude of the electric field exerts repulsive force on hydrophobic residues. Theis force opposes hydrophobic residue aggregation, and therefore alpha helix cannot form. The product of charge, column  3 of Table 1, combined with the summation of charge, relates to the magnitude of electrostatic attractive (or repulsive) force. The summation of hydrophobic character (column  5 of Table 1, ∑_1_^*n*^ *h*_*i*_) is an indication of the net hydrophobic attractive force operating within the region. Using the equalities and inequalities shown in Table 1, very good predictions of secondary structure were obtained (Table 2). It is apparent that the algorithm is sensitive to very small changes in charge and hydrophobic characteristics. The above procedure for determining an alpha helix can be used to determine whether there is an alpha helix region in any portion of interest on the given amino acid sequence. In order to obtain all of the alpha helix regions, the procedure can be repeated by scanning through the entire amino acid sequence. Thereafter, the determination of a beta sheet region can be initiated.

For determining beta sheet, a residue on the amino acid sequence is first selected. If the residue is denoted unstructured (i.e., it is not previously determined to belong to an alpha helix or beta sheet region), the next residue is selected. The procedure is repeated until an unstructured residue  *n*_*i*_  is encountered. A scanning bracket is opened using this unstructured residue as a starting residue, and its next 4 consecutive residues (*n*_*i*+1_  through  *n*_*i*+4_) are queried to determine if they are all unstructured. If the answer is no, the procedure is stopped. Otherwise, a scanning bracket of residues  *n*_*i*_  through  *n*_*i*+4_  is established. Beta sheet determination is then performed based on the summation of the magnitude of charges and the summation of the hydrophobic character of each residue in the 5-residue bracket. For example, such a 5-residue bracket is determined to be a beta sheet when ∑_*i*_^*i*+4^|*q*_*i*_| − ∑_*i*_^*i*+1^ *h*_*i*_ < 0.3 and ∑_*i*_^*i*+1^ *h*_*i*_ > 0.1. The above procedure for beta sheet determination can be repeated for the entire amino acid sequence to obtain all of the beta sheet structures on the sequence.

**Figure 3:**
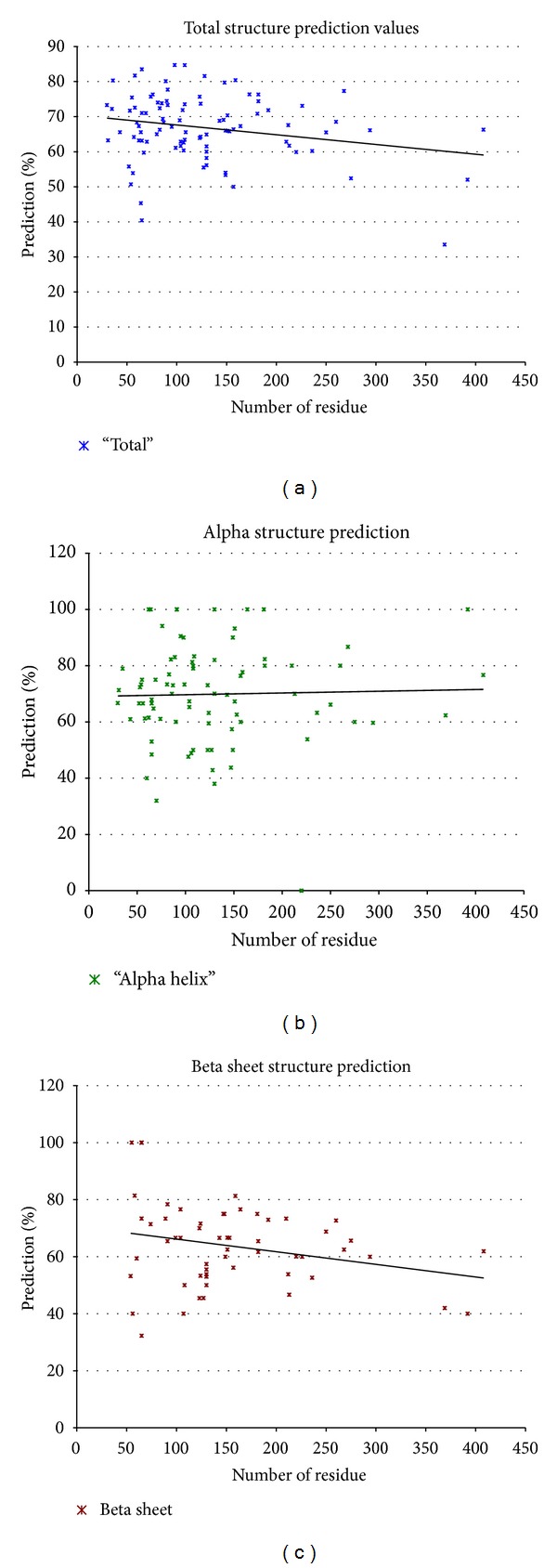
The accuracy of the drift-diffusion model secondary prediction accuracy (percent of proteins tested) versus the size in number of residues. (a) shows the accuracy for total protein core structures versus protein size, (b) shows the accuracy of core alpha helix versus protein size, and (c) shows the accuracy versus protein size. Also shown in each case is the least mean square best fit to the data sets. Data base size ~100 proteins.

Figure 3 shows secondary structure prediction to experiment for a wide cross-section of proteins. The experimental structure was obtained from the Rost and Sander result [22]. The algorithm found essentially all of the secondary structures and correctly determined their character. The accuracy of this procedure has been tested on hundreds of proteins producing accuracies of ~70 ± 9% for alpha helix and ~66 ± 14% for beta sheet identification (residue-by-residue comparison between model and experimental for proper secondary structure assignment). Furthermore, core secondary structure identification was even greater; ~75 ± 7% and ~70 ± 12% for helices and beta sheet, respectively. These are among the most accurate secondary structure predictions to date.

Table 2 compares the accuracy of the KM model (the kinetic model described here) relative to other popular models described in the literature. The kinetic model demonstrated state-of-the-art accuracy in overall secondary structure prediction and excellent alpha helix prediction. The commercial PSIPRED [23] (is based on a statistical approach) also predicted secondary structure regions' size and location extremely well in some cases but in others failed to identify the presence of secondary structure. The kinetic model generally identified the presence of almost all of the structures but the start and end points varied from those of the experiment.

## 4. Tertiary Structure

While various forms of backbone atom tagging have been done previously, for example, in coarse grain models, these approaches differ from that used here. As stated above, the tagging used here involves assigning residue hydrophobicity and charge to the backbone atom bonded to the residue.

In cases where the physical conditions are strongly indicative of a particular secondary structure (e.g., alpha helix), the program directly inserts this secondary structure without wasting computational resource to move atoms to the correct position one-by-one. That is, the secondary structure program is used to identify the strong propensity for secondary structure formation, and where indicated this structure is directly generated by graphical software, for instance, Pymol, and inserted into the tertiary structure. Once a secondary structure is so generated and inserted, it is assumed immutable. For the purpose of continued tertiary structure folding, the inserted immutable secondary structure is tagged with its appropriate summed partial charges and hydrophobicity of each secondary structure region. The portion of the protein not belonging to any determined alpha helix or beta sheet, that is, the unstructured portion, can be built as a linear chain or in an arbitrary physically permissible conformation. During tertiary structure simulation, determined secondary structures inner residues were frozen. During tertiary structure folding simulation, secondary structure regions are treated as one residue.

**Figure 4:**
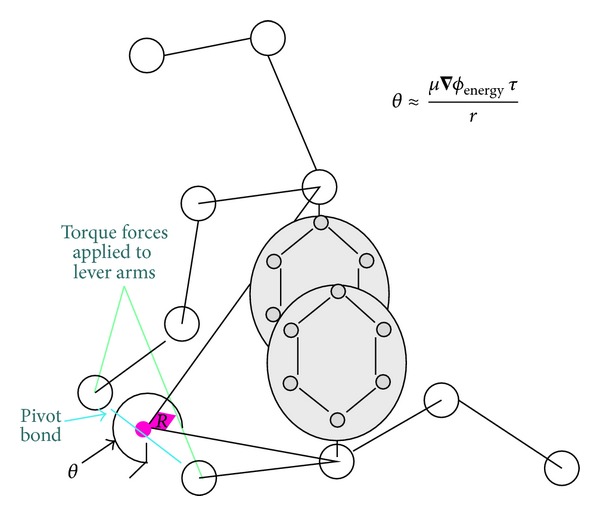
Illustrated is the angle rotation (*θ*  or *R*) about an alpha carbon backbone bond pair induced by the resolved torque operating on the bond. In this figure, each circle represents  *C*_*α*_  atom for each amino acid and big circles illustrate side chains which have strong charges and/or large hydrophobic forces.

As illustrated in Figure 4, the relative motion of one part of a protein relative to the other parts is determined by allowing the alpha carbon bond pair having the largest net torque (sum of actual force and the random effective thermal force multiplied by the appropriate lever arm length) to move in each time step.

**Figure 5:**
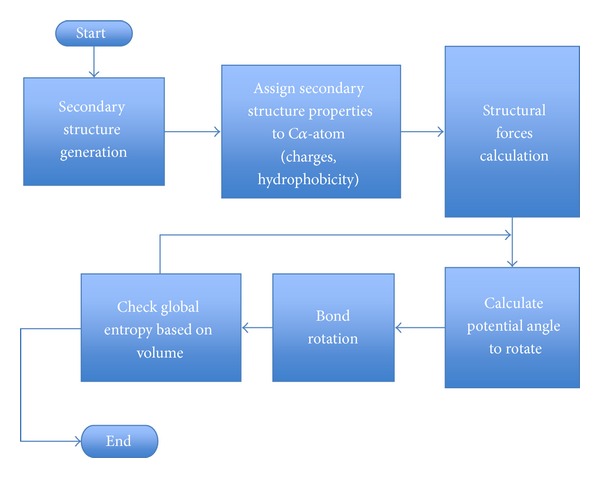
Illustrated is a simplified flow chart of the Markov simulation used in physical-kinetic simulation for tertiary structure.

The protein was allowed to drift and diffuse toward a lower energy in accordance with the procedures described above. The Markov simulation used here is summarized in Figure 5. Starting with linear amino acid sequences, the protein molecule is allowed to drift and diffuse via rotation about the torque rotation angle of pivot bonds as seen in Figure 4. The simulation allows the strongest force (sum of the actual force and the effective thermal force) to operate on the protein for a time period, *t*, using a mobility consistent with (1). In cases where the global energy involves density-dependent entropy, directions that increase entropy proceed with a higher mobility than moves in directions that decrease entropy.

**Figure 6:**
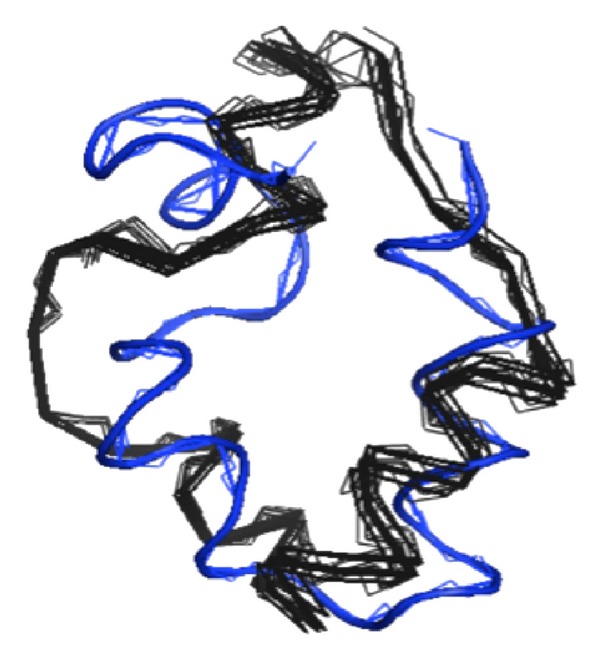
Comparison of a tertiary structure of Villin (1VII) as determined by experiment (NMR structure: blue) with a model generated structure (simulation result: black).

Figure 6 shows a comparison between simulated 3D structure of Villin (1VII) and the experiment. The kinetic model in this example produced near state-of-the-art folded structure with RMSD values for the backbone atoms of test proteins while using less than a minute of desktop computer CPU time. In Table 3, various protein structures ranging from 30 to 157 amino acids were determined using the kinetic model. Average RMSD value for this series relative to experiment was found to be 4.9 ± 1.08 Å.

The largest protein reported here was the human protein tyrosine phosphatome with 320 amino acids. The kinetic model produced a RMSD value of ~8 Å. This value is reasonable relative to other current *ab initio* methods for a protein of this size.

It was found that 2~3% improved RMSD values could be attained using a molecular dynamics structure relaxation. The final MD relaxation step employed no statistical methods or templates. This relaxation employed nominal AMBER10 default parameters for dielectric constant and other parameters. There was no attempt to optimize these parameters. Therefore, these energy relaxations could be carried out quickly (running AMBER on a supercomputer).

**Figure 7:**
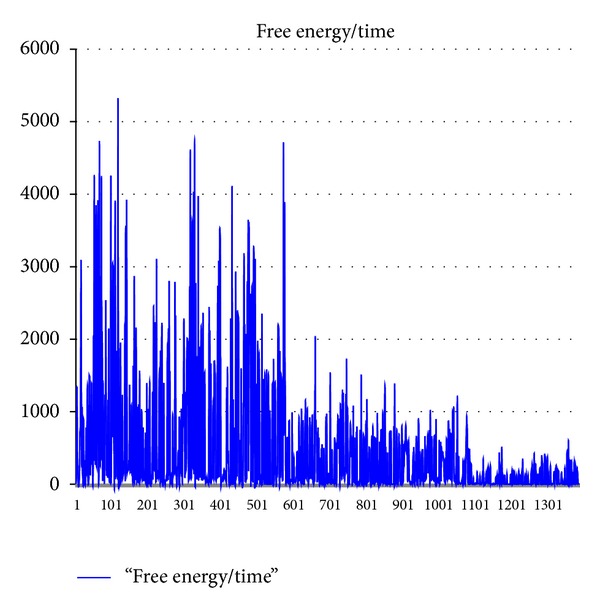
Free energy trajectory (cal/Mol) during Villin simulation.

The energy reduction acting during folding could easily be determined by tracking the force and distance (average) that accompany all folds or motions throughout the simulation.  Figure 7 shows a typical energy versus time trace. The energy was calculated by assuming an arbitrary initial energy and subtracting the work done, *F* · *θ* · *r*_0_, in each time step where *F* is the force applied (the sum of hydrophobic, electrostatic, and thermal forces),  *θ*  is the calculated angle change, and  *r*_0_  is the average lever arm length of the protein. Initially, the energy swings are large and random but as the folding proceeds, these energy fluctuations are diminished. Since the folding is conducted at room temperature, complete cessation of motion does not occur.

## 5. Result

 A highly accurate secondary structure method has been described. The presented physical model predicts secondary structure at least as well as advanced statistical based methods requiring known template structures. The extension of the model to tertiary structure dynamics with near state-of-the-art accuracy has also been described. It was found that thermal and repulsive electrostatic forces are sufficient to prevent unrealistic protein collapse.

This high-speed physical model provides folding trajectory in real time with sensitivity to the environment. Thereby, doors to faster identification of function and a greater understanding of biological pathways are opened. While some statistical methods can match both the speed and accuracy of the kinetic model, it is important to recognize that the relating of structure to function can only be guided by an accurate physical description of the forces that shape proteins.

## 6. Discussion and Conclusion

 The era where protein folding can be tracked as a function of ambient condition without templates or other a priori knowledge has begun. The accuracy of the presented all-physical model rivals the best statistical methods in secondary structure and secondary core structure prediction. The fine-tuning of a secondary structure algorithm can be improved by controlling some environment conditions, for instance, pH. Tertiary structure predictions do not match the best statistical and/or the best molecular dynamics models (when provided with suitable templates). However, the accuracy is amongst the best *ab initio* methods. Further increased accuracy (2~3%) could be obtained using a finishing MD-based energy relaxation step.

For example, the Villin headpiece, a well-studied moderately small protein, has been studied as a function of temperature. Hansmann and coworkers [24–26] achieved very small RSMD values in the range of ~3.0 Å (~1.8 Å for the core region and 3.7 Å for the entire protein) when compared with the NMR determined structure for this protein. In comparison, the all-physical model presented here achieved RMSD values only in the 3.7 Å range.

It is also important to recognize that no model can exceed the accuracy of the measurements used to determine the experimental structure. The Villin headpiece protein experiment has a ~1.8 Å inherent uncertainty. It is often challenging to obtain all of the ambient conditions (including temperature, pH, and process steps that may alter residue charge) used for a given experimental structure determination. On the other hand, the model can be used to track structure change due to small changes in ambient (e.g., pH or temperature). Some MD-based modelings avoid this problem by using a seed or template protein structure obtained under similar experimental conditions. However, such procedure may restrict the modeling applications in real world situations. The kinetic model is expected to continue to improve and become a useful tool for the investigation of protein structure especially where there is little a priori structure knowledge, a need to elucidate the folding pathway, and/or required high speed.

Owing to the speed of the physical model, the protein folding dynamic can be traced. Here, the protein energy is seen to randomly vary initially folioed by secondary formation and finally convergence to a more definite structure with decreasing energy variations as folding precedes. In agreement with experiment, the simulations tend toward definite structure but do not reach complete stasis.

Alternative high-speed computational methods such as EVFold [27] enable widespread access to fast prediction based on statistical analysis of homologous sequences. Even though the two approaches are based on very different paradigms, together and with other contributions there is an emerging and improving ability to predict protein structures without the need to become experts of a particular approach.

To illustrate guidance provided by the physical model, consider the mechanisms by which the chaperone-subunit complex of a diverse group of Gram-negative bacteria [28] gains passage through the *Papc usher* channel at the outer membrane. Here, an approaching chaperone complex triggers a response by which outer membrane usher passage unblocks. Applying the discussed concepts to the blocking structure, it is recognized that the highly hydrophobic blocking structure (since it is comprised of alpha helices) always moves towards lower electric field (second term on the right of (8)). The usher channel interior (beta barrel) is highly charged giving rise to the possibility that the blocking structure is moved to the side of the channel where the electrical field is smallest and whenever an approaching charged structure in combination with the channel charge produces an appropriate small combined electric field. Such insights may lead to new strategies for drug delivery.

## Figures and Tables

**Table 1:** Physical conditions from alpha helix identification.

Case	∑_*i*=1_^*n*^‍*q*_*i*_	Π_*j*=*k*_^*m*^ *q*_*j*_	Other *q*_*i*_	∑_*i*=1_^*n*^‍*h*_*i*_
*n* = 1	0 < *a* < 0.2	>0		
	<−0.5	=0		
			*q*_1_ > 0.9	<−0.3
	1 < *a* < 0.5	<0		

*n* = 3	>1		*q*_2_ ≠ *q*_3_	
	|*a* | <0.5			<−6.0

*n* = 5	0.3 < *a* < 0.5			
	|*a* | >1.0			
			*q*_*i*_ > 0	
			|*q*_*i*_ | >0.6	

*q*_*i*_: partial charge of *t*th residue; *h*_*i*_: hydrophobicity of *i*th residue.

*n*: number of residue.

**Table 2:** Comparison between KM and PSIPRED.

	KM	KM	KM	PSI	PSI	PSI
PDB	% all	% *β*	% *α*	% all	% *β*	% *α*
1hdn	73.8	78.5	82.26	89	88	93
1ubq	76.32	72.73	94.11	82	73	77
1vii	72.2	non	78.95	80	non	100
2nmq	64.18	65.38	non	60	54	non
1pba	74.04	non	73.33	68	non	70
1aps	84.7	80	90	80	83	87
1aey	67.3	77.8	non	44	35	non
1coa	45.31	71.05	61.53	81	60	92
1fkb	61.61	72.97	100	88	90	100
1mjc	71.01	80.64	non	69	70	non
1nyf	59.73	40	70	62	54	0
1pks	65	53.57	64.75	59	48	0
1shg	63.2	61.23	non	42	25	non
1srl	65.56	75	100	53	37	0
1ten	74.44	68	non	80	85	non
1ycc	63.4	58.58	60.01	51	0	53
2ci2	66.26	64.34	79.84	47	28	0

Avg	67.59	67.99	79.98	66.76	55.33	51.69

KM means the developed model and PSI means PSIPRED, which is developed by the University of London. Other names mean the percentage of prediction of total structure, alpha-helix, and beta-sheet with respect to two different methods. To compute prediction rate, NMR structure of each protein was used.

**Table 3:** RMSD result for tertiary structures.

Proteins	No. RES	Score for 2D	RMSD of 3D
2MHU	30	73.3	3
1VII	35	72.2	4.8
1CBH	36	75.3	4.3
3RNT	54	61.65	4.1
1DUR	55	74.45	3.7
1OVO	56	53.9	5.8
1BW6	56	70	6.1
2NMQ	57	64.18	5.8
2UTG	70	62.85	3.8
1UBQ	76	76.32	5.4
2PCY	99	61.1	4
5CYT	103	68.94	4.7
7RSA	124	73.7	5.1
1PAB	127	74.04	4.2
2CCY	128	81.56	6.3
2SNS	149	54.02	6.7
1AAQ	157	73	6.2

AVG	83.06	69.94	4.94

A tertiary structure prediction result based on the kinetic model. RMSD values are based on each backbone atom position comparison for tertiary structure prediction. Also a score of 2D means the matching percentage for the secondary structure determination.
